# Effects of Tibolone on the Central Nervous System: Clinical and Experimental Approaches

**DOI:** 10.1155/2017/8630764

**Published:** 2017-01-16

**Authors:** Rodolfo Pinto-Almazán, Julia J. Segura-Uribe, Eunice D. Farfán-García, Christian Guerra-Araiza

**Affiliations:** ^1^Unidad de Investigación del Hospital Regional de Alta Especialidad de Ixtapaluca, Carretera Federal México-Puebla km 34.5, Pueblo Zoquiapan, Municipio de Ixtapaluca, MEX, Mexico; ^2^Institute for the Developing Mind, Children's Hospital Los Angeles, Los Angeles, CA, USA; ^3^Department of Pediatrics, Keck School of Medicine, University of Southern California, Los Angeles, CA, USA; ^4^Escuela Superior de Medicina, Instituto Politécnico Nacional, Plan de San Luis y Díaz Mirón, 11340 Mexico City, Mexico; ^5^Unidad de Investigación Médica en Farmacología, Hospital de Especialidades, Centro Médico Nacional Siglo XXI, Instituto Mexicano del Seguro Social, Av. Cuauhtémoc 330, Col. Doctores, Mexico City, Mexico

## Abstract

Hormone replacement therapy (HRT) increases the risk of endometrial and breast cancer. A strategy to reduce this incidence is the use of tibolone (TIB). The aim of this paper was to address the effects of TIB on the central nervous system (CNS). For the present review, MEDLINE (via PubMed), LILACS (via BIREME), Ovid Global Health, SCOPUS, Scielo, and PsycINFO (ProQuest Research Library) electronic databases were searched for the results of controlled clinical trials on peri- and postmenopausal women published from 1990 to September 2016. Also, this paper reviews experimental studies performed to analyze neuroprotective effects, cognitive deficits, neuroplasticity, oxidative stress, and stroke using TIB. Although there are few studies on the effect of this hormone in the CNS, it has been reported that TIB decreases lipid peroxidation levels and improves memory and learning. TIB has important neuroprotective effects that could prevent the risk of neurodegenerative diseases in postmenopausal women as well as the benefits of HRT in counteracting hot flashes, improving mood, and libido. Some reports have found that TIB delays cognitive impairment in various models of neuronal damage. It also modifies brain plasticity since it acts as an endocrine modulator regulating neurotransmitters, Tau phosphorylation, and decreasing neuronal death. Finally, its antioxidant effects have also been reported in different animal models.

## 1. Introduction

According to the definition of K. Jain, an agent or compound that prevents neuronal death in processes that include injury or ischemia, protects against neurotoxins or neurodegeneration, and slows or halts the progression of neuronal degeneration should be considered as a neuroprotectant [1].

Experimental evidence has shown the neuroprotective effects of sexual hormones (estradiol and progesterone) in different animal models, such as the ovariectomized rodent, the stroke model, and the traumatic brain injury [2–5]. However, many studies have questioned the use of estrogen as neuroprotectants in humans and emphasized the risk of breast and endometrial cancer and stroke [6–9].

### 1.1. Hormone Replacement Therapy

Due to the increase in life expectancy, women live for approximately 30 years or more after menopause. This fact has increased the interest of knowing the effects of the absence of estradiol and progesterone in the central nervous system (CNS) [10–12].

Hormone replacement therapy (HRT) involves the administration of sexual hormones to replace depleting hormone levels in women. HRT might contain estrogen alone or combined with a progestogen for endometrial protection. HRT with estrogen (estradiol, 17*β* estradiol, estrone, or conjugated equine estrogen) can be oral, intravaginal, or transdermal. Progestogen can be oral, transdermal, or delivered via an intrauterine device [13].

The main objective of HRT is to treat vasomotor symptoms and to prevent osteoporosis [6]. Those effects are related to the plasmatic decrease of estrogen in peri- and postmenopausal women. Furthermore, it has been described that estrogen replacement therapy (ERT) diminishes the risk of neurodegenerative diseases, including Alzheimer's disease, because this hormone acts on several neuronal pathways [14]. ERT and combined estrogen-progestogen therapy (CEPT) have been recognized as effective treatments to alleviate menopause symptoms. Unfortunately, they increase endometrial and breast cancer risk, respectively [5, 6].

### 1.2. An Overview of TIB

Another strategy to achieve the estrogen/progesterone balance desired on HRT target tissues is the use of a steroid with combined estrogenic/progestogenic activities in vivo [10]. Tibolone (TIB) is a synthetic steroid with estrogenic, androgenic, and progestogenic actions that can be used as HRT. Unlike estrogen, TIB has no estrogenic activity in endometrial and breast tissues but has effects in counteracting perimenopause symptoms [15, 16].

TIB is a selective tissue estrogenic activity regulator (STEAR) that has been used as a first-choice treatment to alleviate climacteric symptoms and to prevent osteoporosis [15]. Although TIB shows a lower affinity for estrogen, androgen, and progesterone receptors in comparison with their respective ligands, it presents estrogenic effects in bone tissue, vagina, and brain; progestogenic effects in breast tissue and endometrium; and androgenic effects in the liver [15–17].

To date, the use of TIB is registered in approximately 90 countries, such as Australia, China, Korea, India, and Spain, for the alleviation of climacteric symptoms, and in 45 other countries for the prevention of osteoporosis [18–20]. TIB, the active ingredient in Livial®, was synthesized for the first time in the late 1960s and its bone loss preventive properties were tested in the early 1970s. In the first clinical study in women, Lindsay et al. demonstrated the positive effect of TIB in bone loss [21]; additionally, it seemed that although TIB alleviated climacteric symptoms, there were no estrogenic effects observed in the endometrium [22]. These findings initiated new research in the field of menopausal symptoms and prevention of osteoporosis [15, 23].

**Figure 1:**
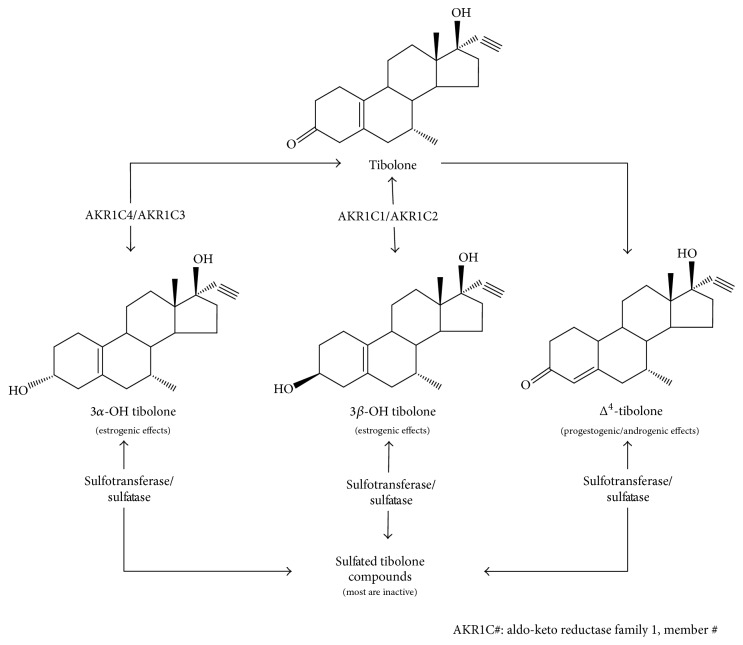
Chemical structure of TIB and its metabolites. After oral intake, TIB is metabolized by aldo-keto reductase (AKR) family members, which are a superfamily of NADPH-dependent oxidoreductase enzymes also called hydroxysteroid dehydrogenases (HSD). From this reaction, three different metabolites are originated: 3*α*-hydroxy tibolone (3*α*-OH TIB) and 3*β*-hydroxy tibolone (3*β*-OH TIB) in their sulfated inactive forms and Δ^4^-tibolone (produced from them or directly from TIB). Hydroxysteroid dehydrogenase metabolites *α* and *β* have a high affinity for ER, while Δ^4^-tibolone has an affinity only for PR and AR. The tissue-specific activity of TIB will depend on the interaction of two main physiological mechanisms: biochemical mechanism (TIB metabolism) and genetic mechanism (through the interaction with the steroid receptor). Therefore, the action of TIB will also depend on its metabolism at the targeted organ, as well as the interaction of its metabolites with the receptors to which they bind [24].

After ingestion, TIB is rapidly metabolized in the liver and the small intestine into 3*α*-hydroxy-TIB (3*α*-OH-TIB) and 3*β*-hydroxy-TIB (3*β*-OH-TIB) in their sulfated inactive forms. A third compound, delta 4-TIB isoform (Δ^4^-TIB), is formed from them or directly from TIB [17]. Due to a very fast metabolism in the small intestine, TIB and Δ^4^-TIB plasmatic levels are almost undetectable. Hydroxylated metabolites reach their peak between 60 and 90 min, and their plasmatic half-life is about seven hours (Figure 1) [24].

### 1.3. Mechanisms of Action of TIB

Although TIB can bind to all three hormone receptors mentioned above, its effects are carried out mainly by its metabolites because of TIB's rapid absorption and distribution after its oral intake [24]. Both 3*α*-OH-TIB and 3*β*-OH-TIB isomers have a high affinity for ER, while Δ^4^-TIB has more affinity for AR and PR. Therefore, the biological effect of TIB is determined directly by the enzymatic characteristics and chemical capacities, as well as ER, PR, and AR levels in each tissue [22].

In 2007, Verheul et al. quantified TIB and its metabolites concentration in different brain regions from ovariectomized (OVX) monkeys treated with TIB for 36 days. They reported high levels of estrogenic metabolites of TIB in hypothalamus, hippocampus, cerebellum, cerebral cortex, and brainstem compared to serum levels. These elevated levels of estrogenic metabolites in different regions of the CNS explain TIB effects on brain function [25].

For several years, TIB has been administered for the treatment of symptoms and to prevent osteoporosis in postmenopausal women. However, the mechanisms through which TIB exerts its effects in the CNS are unknown. Controversy exists between the beneficial and adverse effects of the use of TIB in the long term. Therefore, the aim of this review was to address the results from controlled clinical trials as well as from basic experimental studies performed to analyze effects of TIB on the CNS and to suggest the role of TIB as a neuroprotectant.

## 2. Methods

This manuscript included the search of controlled clinical trials from MEDLINE (via PubMed), LILACS (via BIREME), Ovid Global Health, SCOPUS, Scielo, and PsycINFO (ProQuest Research Library). Language restriction was applied to English. All searches were performed from 1990 to September 2016 and included the controlled vocabulary indexed on databases as well as keywords. Terms used on Medical Subject Heading (MeSH) were “Tibolone”; “Hot Flashes”; “Affects”; “Libido”; “Memory”; “Cognitive Deficits”; and “Central Nervous System”. For Descriptors in Health Sciences (DeCS), the terms used were “Hot Flashes”; “Affect”; “Libido”; “Memory”; “Neurocognitive Disorders”; and “Central Nervous System”. The Boolean operator “AND” was used to perform a broad range of combinations in databases and find all relevant studies. This manuscript also included experimental studies obtained with the MeSH terms “Tibolone” AND “Brain.”

## 3. Results

### 3.1. Effects on Climacteric Symptoms (Hot Flashes, Mood, and Libido)

Peri- and postmenopausal women frequently present climacteric symptoms such as vasomotor symptoms (hot flashes, diaphoresis), urinary tract symptoms, sleep disturbances, mood changes, or sexual problems (vaginal dryness, loss of libido, and dyspareunia) [26, 27].

Hot flashes have been attributed to the dysfunction of several neurological pathways during menopause. Some of them are the hypothalamic thermoregulatory threshold to circulating estrogen, destabilization of the thermoregulatory benchmark through an alteration of serotonin hyperthermia receptors (5-HT2A) versus hypothermia receptors (5-HT1A) [28], and a reduction in peripheral thermal neutrality (skin) [29]. Estrogen modulates thermoregulatory control at both central and peripheral levels [30].

Garefalakis and Hickey reported that the effects of TIB on sweats, hot flashes, and bone were similar to those observed for ERT [31].

TIB decreases the frequency and intensity of hot flashes in a dose-dependent manner. The optimum dose is 2.5 mg, with a significant benefit observed after four weeks of use and maximum effect after 12 weeks. After 12 weeks of treatment with 1.25 mg or a greater dose, 86% of women had no or very mild symptoms compared to 55% treated with placebo. Women with more severe symptoms had a previous benefit (four weeks) with a daily 1.25 mg dose, but more women abandoned the treatment (10%) because the therapeutic effect seemed insufficient compared to the 1% of women that stopped the daily 2.5 mg treatment [32].

No evidence shows that menopause causes depression. However, climacteric changes and symptoms affect the psyche [33, 34]. Around 20% of perimenopausal women are depressed, and it has been suggested that mood alterations could be related to the biological changes that occur during menopause. Depressed mood is linked to low levels of estrogen. The central role of estrogen in the brain, especially in areas with many receptors such as the limbic area, suggests the hypoestrogenism as an etiology [33, 34]. On learning the properties of TIB to improve depressive mood in postmenopausal women, Berlanga et al. designed a study to assess whether TIB could have a synergistic effect with fluoxetine for the treatment of the major depressive disorder (MDD). For the study, 31 postmenopausal patients with a MDD were divided into two groups of treatment: group 1 received fluoxetine plus TIB (16 patients), and group 2 received fluoxetine plus placebo (15 patients) for eight weeks. In the end, both treatments were well tolerated, but treatment with TIB plus fluoxetine did not produce a more robust antidepressant response than fluoxetine alone [35].

TIB has a beneficial effect on the mood of climacteric women during and after menopause. A double-blind crossover study of 256 postmenopausal women receiving TIB (2.5 mg/day) or placebo found a significant improvement in mood in the TIB treated group compared to the placebo group [36]. In addition, an open cross-study in 82 women taking TIB also demonstrated improvements in mood and sense of well-being [37]. An explanation for this improvement is provided perhaps by the specific tissue effect that TIB exerts in the brain and particularly on the increase of endorphins. It has been shown that *β*-endorphin levels decrease significantly after menopause [38]. This decrease can be prevented after TIB treatment. A study of 30 postmenopausal women who were treated for six months with TIB showed that *β*-endorphin levels returned to premenopausal levels approximately eight weeks after the beginning of the treatment, unlike the placebo group in which endorphin levels did not change. In this same study, TIB demonstrated a similar effect on mood as the CEPT regime [39].

In a study by Albertazzi et al., memory, libido, and mood were compared in postmenopausal women treated with 2.5 mg of Livial (TIB) or treated with Kliogest® (1 mg norethisterone acetate (NETA) and 2 mg of estradiol) for six months. They found that both treatments improved libido while mood did not change with either of them [40]. Moreover, Garefalakis and Hickey reported that TIB improved libido more effectively than ERT [31].

Also, the effects of TIB on mood and libido have been studied in surgically menopausal women. Gupta et al. and Somunkiran et al. demonstrated that TIB may improve mood, libido, and climacteric symptoms better than DHEA (dehydroepiandrosterone) and 17*β*-estradiol alone and is almost as useful as conjugated equine estrogen (CEE) but with fewer side effects after more than six months of use [41, 42].

On the other hand, a study on the cognitive effects in women after ten years of treatment with different HRT and after being subjected to a test of mild stress showed that women who did not receive TIB had greater anxiety symptoms compared to those who received treatment [43].

Although sexual desire and arousal normally decline with aging, many peri- and postmenopausal women indicate a decrease in libido as an important part of this process perhaps due to central origin problems or secondary or peripheral problems, such as vaginal atrophy or urethral syndrome [44]. Several reports indicate that TIB improves desire and sexual function in postmenopausal women, and it is as effective or more effective than CEPT treatment [45–47]. The increase in sexual desire induced by TIB may be due to estrogen-androgen effects and the low activity of the sex hormone binding globulin (SHBG) [48] or because changes in sexual function are correlated with the increase in both free E2 and testosterone (T) rates [46]. Postmenopausal women with sexual dysfunction indicated an improvement according to the Rosen Index of Female Sexual Function (RIFSF) [45] and even higher scores than women with continuous transdermal CEPT (50 mg/140 mg) [44]. Some factors which may contribute to the above findings are that TIB improves vaginal pulse amplitude (measured vaginal blood flow) [48], normalizes the vaginal maturation index, and alleviates the symptoms of atrophic vaginitis [49] in postmenopausal women.

### 3.2. Effects on Cognitive Deficit

Another physiological aspect affected after menopause is memory. In this respect, postmenopausal women treated with estradiol improve data collection, memory, and other aspects of cognitive function, including cognitive impairment. These results correlate directly with the presence of bioavailable plasma E2 [50]. In recent years, several studies on TIB effects in memory and cognition have been conducted [40, 43, 51–58].

As mentioned above, Albertazzi et al. reported that postmenopausal women treated with 2.5 mg of TIB for six months had improved long-term semantic memory similarly to women receiving CEPT (Kliogest) [40]. This effect might be explained because TIB decreases the SHBG, and this could increase the bioavailability of E2 and T [59].

After ten years of treatment with different HRT, a study on the cognitive effects in women showed that participants who received TIB had a better response in semantic memory (memory for facts) tests evaluated in a task of generating categories but did not differ in episodic memory (memory for events) tests. Finally, it was reported that the TIB group fared worse on a sustained attention task and a planning task, both tasks associated with frontal lobe function [43].

In another study, it was reported that the administration of TIB reverses damage on cognition that is caused by leuprolide acetate and improves mood and the quality of life of patients receiving releasing hormone (GnRH) agonists for the treatment of symptoms of uterine leiomyomas [56].

The effect of long-term use of TIB has also been studied in different animal models. In OVX young, adult, and aged rats, TIB improved memory in a step-down-type passive avoidance test. However, in this study, sham and OVX rats, as well as young OVX rats treated with a dose of 0.5 mg/kg of TIB, had a worse performance in the water maze compared with control animals [53]. In a model of menopause (18 weeks of OVX), high doses of TIB (1 and 10 mg/kg) improved learning by increasing T-shaped maze latencies compared to those treated with vehicle [54].

In 2014, it was reported that chronic treatment with TIB for different periods of time prevented cognitive deficits in short- and long-term memory in a model of oxidative stress induced by chronic ozone exposure [60].

### 3.3. Neuroplasticity and Neurotransmission

Although cognitive aspects and neuroprotection have not been fully elucidated, studies in animals and humans suggest that TIB may be useful against certain types of neuronal damage. Among the damage-inducing factors that have been investigated are menopause [40–43], drug administration [56], long-term OVX [55, 58], oxidative stress [57, 60], and aging [54]. Furthermore, TIB has been proposed as an endocrine modulator [52] and regulator of neurotransmitters [55].

Regarding neuroprotection, the administration of TIB quickly attenuated the response of GABA-B receptor agonist, baclofen, in a study of guinea pig hypothalamic neurons [61]. Moreover, Espinosa-Raya et al. reported an increase in learning in a model of menopause with a chronic treatment of TIB at high doses (1 and 10 mg/kg). These results were correlated with a decrease in the content of choline acetyltransferase (ChAT) with increasing doses of TIB, whereas the content of tryptophan hydroxylase (TPH) increased with doses of 1 and 10 mg/kg of TIB. These data suggest the effects of TIB in the modulation of the cholinergic and serotonergic systems [55].

These results agree with those of Gibbs et al. who reported significant reductions in both ChAT and acetylcholinesterase (AChE) in the medial septum/diagonal band of Broca. This effect was dose-dependent in OVX monkeys treated for two years with TIB [51]. In another study, it was reported that ozone exposure decreases the content of ChAT and acetylcholine, while TIB maintains normal levels of this enzyme and this neurotransmitter in the hippocampus [60].

Other studies have reported that chronic treatment with 0.5 mg/kg of TIB in OVX adult rats significantly decreased the content of hyperphosphorylated Tau protein and increased dephosphorylated Tau. These changes were correlated with the increased form of phosphorylated GSK3 in the hippocampus and cerebellum. These results indicate that TIB can modulate the phosphorylation of Tau and thus may help to prevent the effects observed in certain tauopathies, such as Alzheimer's and other dementias [3, 4].

In 2012, a study reported a greater intensity of immunohistochemical staining for glial fibrillary acidic protein (GFAP) and c-Fos in cerebral cortex and hippocampus of TIB treated female rats compared to control animals. As the primary function of GFAP is protecting the internal organization of astrocytes and c-Fos is a neuronal activity marker, the increased expression of both indicates the beneficial effects of TIB in these two aspects [62].

Another study reported that neuroprotective effects of TIB had been observed with a 1 mg/kg dose treatment to reduce neuronal death in hippocampal CA3 region and cognitive and motor damage in a rodent model of oxidative stress induced by chronic ozone exposure for different periods of time [60].

On the other hand, TIB showed protective actions against glucose deprivation in a human astrocyte cell model (T98G cells) [63]. Furthermore, as TIB metabolites activate ER*α* and ER*β* in human astrocytes [64], it has been suggested that some of TIB neuroprotective effects could be mediated in these cells. Nonetheless, further studies need to be undertaken to determine whether TIB has effects on neuroinflammation and reactive astrogliosis [65].

### 3.4. Oxidative Stress

TIB effects on oxidative stress have been studied in various animal models [54, 57, 66]. In a recent study, Stark et al. observed that TIB showed no significant antioxidant capacity, but most of its active metabolites did [67]. These results indicate that TIB metabolites are those that perform the antioxidant effects. In 2008, Aguiar et al. reported that a 12-week TIB treatment at doses of 0.5 and 1 mg/kg/day increased the overall antioxidant capacity in the cerebral cortex and hippocampus of OVX adult rats. However, this effect was not observed in either young or old rats, in which it seemed to be diminished when compared to OVX untreated and sham groups.

Furthermore, TIB reduced lipid peroxidation (LPO) levels in the hippocampus of young rats compared to that of OVX rats without treatment [54]. In another study, it was reported that TIB did not reduce lipid peroxidation in the hippocampus but recovered pyramidal neuron spine pruning of CA1 hippocampus produced by OVX in rats [66]. It should be mentioned that OVX is not a suitable model of oxidative stress since important changes around stress were not observed in these studies. However, beneficial effects were found with TIB treatments in both studies.

It has been reported that TIB treatment (1 mg/kg/day) before chronic ozone exposure prevented the increase of oxidative stress markers such as 4-hydroxynonenal and nitrotyrosine in an in vivo oxidative stress model [57], specifically in the CA3 region of the hippocampus [60].

### 3.5. Stroke

Stroke incidence increases substantially after menopause. It is the third cause of death and the leading cause of disability in industrialized countries. It is caused by disruption of blood flow—and a consequent oxygen supply—to any region of the brain [68]. Different studies suggest that there are sex differences regarding stroke. Women have a higher prevalence of stroke than men, and the risk increases rapidly with age in postmenopausal women [68, 69]. One of the major inconveniences of the use of HRT is the apparent increased risk of stroke. Data from the Women's Health Initiative (WHI) showed that the use of CEE and combined CEE/MPA (medroxyprogesterone acetate) therapy increased this risk. However, there was no risk increase in the group of 50–59-year-old women [69]. Furthermore, in a model of focal cerebral ischemia in OVX rats treated with 200 mg administered subcutaneously, TIB reduced infarct volume caused by ischemia. In this study, the same treatment had no effect on male rats [70].

Long-term Intervention on Fractures with Tibolone (LIFT) study reported an increased risk of stroke; therefore, TIB therapy was suspended by February 2006. This study evaluated 4,538 postmenopausal women of 65 to 85 years of age (mean age of 68 years) with the hypothesis that TIB would reduce the number of vertebral fractures. All women had a *T* score of bone mineral density of −2.5 or less at the hip or spine. TIB was administered at doses of 1.25 mg achieving 45% reduction in vertebral fractures and 26% of nonvertebral fractures compared with placebo and a reduced risk of breast and colon cancer. However, even when the expected results were obtained, the study was suspended because a twofold risk increase of stroke or ictus was observed. It should be noted that this study was conducted several years after menopause of these women [18].

## 4. Discussion

Before and after LIFT study, several studies (LISA, OPAL, THEBES, STEP, and LIBERATE) have supported the use of long-term TIB. These long-term studies (from 24 weeks to three years) were designed to evaluate different characteristics of TIB compared to placebo treatment as well as with other commonly used hormonal therapies on postmenopausal women.

In LISA, OPAL, and THEBES studies, TIB proved to be as effective as CEPT in preventing bone loss, reducing fractures as well as improving libido and sexual function. Also, TIB was shown to be as safe as CEPT by not presenting any difference on increasing the risk of adverse effects, such as stroke, hyperplasia, or carcinoma, in comparison with the CEPT or placebo treatment. It should be emphasized that these studies were conducted in women <60 years of age (mean age of 56 years for LISA, 58.77 years for OPAL, and 54.5 years for THEBES).

In contrast, the mean age of the LIFT trial was 68.3 years. A possible explanation for the increased risk of stroke present in the LIFT study can be because these women began TIB treatment several years after menopause. When the drug is given during perimenopause, it has been shown to have a comparable risk of stroke like other conventional therapies [71].

It should be noted that in the STEP study (mean age of 66 years), in which the primary objective was to compare the effects of TIB and raloxifene on lumbar vertebrae bone mineral density (BMD) in osteopenic women, TIB showed a comparable risk of stroke like other conventional therapies. This study was carried for two years and demonstrated that TIB prevented postmenopausal bone loss, increased BMD, and showed a trend towards an improvement in the quality of life and sexuality when compared with raloxifene [72].

Therefore, an important consideration to avoid the increased risk element might be the starting point of the therapy, which should start during perimenopause or just past menopause and should end after five years or before the age of 60 years. In 2007, Lobo analyzed the incidence of stroke in HRT users (CEE and MPA) versus nonusers in several studies. In this analysis, the rate of stroke in 50–59-year-old white women (0.6–0.8/1000 women/year) increased to almost 2/1000 women of 60 to 64 years of age and 4.2/1000 women at the age of 65–74 years. In the WHI study, stroke mostly occurred in older women, and the increased risk of stroke in the entire population using CEE/MPA and CEE was 1.41 (95% confidence interval (CI) of 1.07–1.85) and 1.39 (95% CI of 1.10–1.77), respectively. Nevertheless, the risk was not increased in the 50–59-year-old group for CEE (1.09 (95% CI of 0.59–2.21)). In conclusion, the author suggested that HRT can be used in younger (50–59 years) normotensive postmenopausal women, particularly when lower doses are prescribed soon after menopause [69]. A year later, Cummings et al. came to the same conclusion when analyzing the long-term effects of TIB in older postmenopausal women. They concluded that TIB should not be used in elderly women or women with strong risk factors for stroke, such as hypertension, diabetes, atrial fibrillation, and smoking [18].

Nevertheless, none of the studies mentioned above reported any increase regarding the brain condition.

## 5. Concluding Remarks

**Figure 2:**
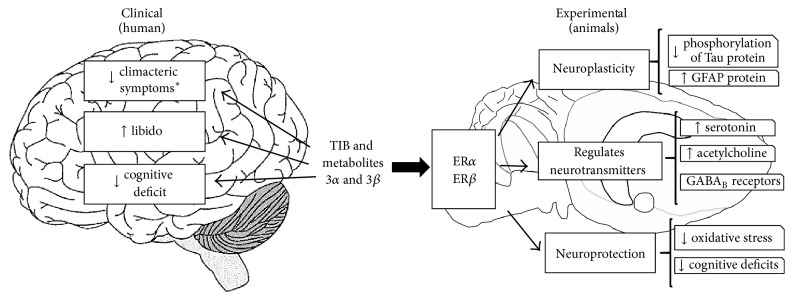
Clinical and experimental approaches of TIB in the central nervous system. ^*∗*^Climacteric symptoms: vasomotor symptoms, mood, and well-being. ↑: increase; ↓: decrease (see [4, 7, 10, 11, 18–22, 29–31, 34–67]).

ERT and CEPT are effective treatments to relieve menopause symptoms but unfortunately increase cancer incidence. TIB is a synthetic molecule that eliminates menopause symptoms such as hot flashes and night sweats and improves mood and libido. It also decreases the cognitive deficit, which can be attributed to the high levels of estrogen metabolites binding to ER in different brain regions. Moreover, TIB modifies brain plasticity as it acts as an endocrine modulator of neurotransmitters and Tau phosphorylation and increases neuronal activity. It also has antioxidant effects in different animal models (Figure 2).

The beneficial effects of TIB are facilitated due to the characteristics that are desirable in a neuroprotective agent (e.g., being orally active for ease of administration over extended periods of treatment, daily doses with therapeutic concentrations, compatibility for coadministration with other drugs, and acceptable safety profiles with a good risk/benefit ratio [1]).

Based on the analysis in this manuscript, we suggest that TIB has neuroprotective effects that could prevent some neurodegenerative diseases. Therefore, TIB may be considered as a neuroprotectant drug with most of the desirable characteristics.
